# Comparative Evaluation of Arthritic Changes in Post-operative Patients With Anterior Cruciate Ligament (ACL) Injuries: A Study of Exercising Versus Sedentary Individuals

**DOI:** 10.7759/cureus.77264

**Published:** 2025-01-11

**Authors:** Varun Kshirsagar, Trupti Yadav

**Affiliations:** 1 Krishna College of Physiotherapy, Krishna Vishwa Vidyapeeth, Karad, IND

**Keywords:** anterior cruciate ligament reconstruction, arthritic changes, exercise, physical activity, sedentary lifestyle

## Abstract

Background: Anterior cruciate ligament (ACL) injury has become quite a common injury affecting the knee in young and middle-aged individuals. ACL reconstruction is a common surgical intervention indicated after a grade 3 rupture. Meanwhile, early arthritic changes start to appear after five years post-ACL reconstruction. Increased knowledge about regular exercise after ACL reconstruction in the sedentary population can decrease or reduce the chances of having early arthritic changes in the knee. Therefore, a study is needed to find the prevalence of arthritic changes in the exercising and sedentary postoperative ACL injury patients.

Materials and methods: The population was divided into sedentary and exercising populations. The exercising population was further divided into low, moderate, and high-intensity exercises based on the target heart rate range and maximum heart rate. Radiographs just before surgery or immediate post-operative versus recent radiographs (after five years) were used to assess the knee joints of exercising and sedentary populations by using the Kellgren and Lawrence classification (KLC) to measure the severity of knee osteoarthritis.

Results: In the exercising population, only 4(9.52%) of the individuals were found to have arthritic changes in their knee, whereas, in the sedentary population, 10(18.51%) of the individuals had arthritic changes in their knee post-ACL reconstruction after five years. Lack of regular exercise after ACL reconstruction showed early arthritic changes compared to those who were physically active, indicating the impact of physical activity.

Conclusions: This study concludes that a greater number of the sedentary population had arthritic changes in their knee joints post-surgery than those who were physically active, highlighting the significance of regular exercise.

## Introduction

A traumatic development known as an anterior cruciate ligament (ACL) injury might result in severe functional impairment and the inability to engage in high-level sporting events. For patients with symptomatic ACL deficiency, ACL reconstruction is the preferred method of treatment that can help them fully recover. In India, there are 68.6 ACL tears per 100,000 individuals per year, and over 125,000 ACL repairs are performed. The ACL is a thick band of connective tissue that runs from the tibia to the femur [1]. The ACL is a key structure in the knee joint as it resists anterior tibial translation, internal rotation loads, and valgus angulation.

There are two types of ACL injuries: contact and non-contact. The most frequent mechanism is an excessive valgus movement triggered by a force applied to the lateral side of the knee [2]. The most frequent non-contact injury is caused by an external rotational mechanism that causes the tibia to rotate outward on the planted foot. Excessive forceful hyperextension of the knee is the second most prevalent noncontact mechanism [3,4]. Anteroposterior and rotational instability, as well as functional impairment, are caused by ACL insufficiency. Due to their smaller knee flexion angle, greater knee valgus angle, greater ground reaction forces, and greater knee extension moment during the landing of specific sports activity, women are more likely than men to have an ACL injury [5,6].

Since a torn ACL has a limited ability to recover, surgical restoration is typically advised to maintain knee stability, especially in young and active people. When it comes to autografts, the semitendinosus-gracilis tendon autograft (hamstring tendon) and the patellar tendon autograft are the two most common forms [7].

Osteoarthritis (OA) of the knee is a common degenerative joint disease characterized by persistent pain and functional impairment. Almost 80% of OA cases worldwide are caused by knee OA. The incidence of knee OA for people 20 years of age and older was 203 per 10,000 person-years (95% CI: 106-331) globally. Similarly, the global yearly incidence of knee OA in adults (20 years of age and older) is estimated to be 86.7 (95% CI: 45.3-141.3) million in 2020 [8]. Radiological alterations such as loss of joint space, subchondral bone sclerosis, and osteophyte formation suggest significant clinical changes. The prevalence of death among OA patients is generally greater than in the general population. In fact, people with arthritis, especially those with knee and hip OA, have been reported to have an increased risk of death from comorbid conditions like diabetes, cancer, and cardiovascular diseases [9,10].

ACL tears can have numerous adverse effects on the joint. The injuries cause the synovial fluid's delicate chemical balance to be upset, which promotes joint deterioration. Inflammatory markers such as Interleukin-1 (IL-1) and tumour necrosis factor-alpha (TNF-α) increase due to joint injury. Despite ACL reconstruction, the surgical process exacerbates joint injury and prolongs the inflammatory healing period. These pro-inflammatory cytokines are known to inhibit cartilage regeneration and have been linked to cartilage degradation. One possible reason for the emergence of osteoarthritis after ACL surgery is the "first impact" idea, which suggests that a first injury to the knee starts a cartilage-degrading process that eventually leads to osteoarthritis [11].

In light of this, the majority of research on postoperative ACL injuries concentrates on immediate objectives, such as functional abilities and a return to athletics. Through this study, we especially looked at the risk of arthritic alterations in the knee in both sedentary and active individuals. Although there are some controversies regarding studies proving whether isolated ACL reconstruction can cause or alter the progression of OA, other studies have shown a higher risk of OA occurring in the long term [12,13]. Exercise is beneficial for maintaining physical fitness and general health, but high-impact exercises that involve the knee may pose a risk to the reconstructed joint. We can offer insights into the possible advantages and disadvantages of high-impact activities leading to arthritic changes in the reconstructed knee joint by carrying out this study. The same holds true for leading a sedentary life. Individuals who do not engage in physical activity following ACL surgery may also develop knee arthritis. Obesity and sedentary lifestyles are strongly related because most of them include little physical activity, which can especially cause increased joint load following an ACL surgery and result in knee OA.

## Materials and methods

This study assessed whether physical inactivity can cause arthritic changes in post-operative ACL injury patients. The study was conducted in Karad. Certification was taken from the ethics committee of Krishna Vishwa Vidyapeeth (protocol number-155/2023-2024). Then, permission was obtained from the authorities and the ethical committee. Patients were selected according to inclusion and exclusion criteria. Informed consent was obtained, and data was collected. The duration of the study was one year. 

Local Orthopaedic hospitals in and around Karad were visited to collect samples. Some of the hospitals were not allowed to share samples due to privacy policies. At the same time, the rest gave the patient’s contact numbers upon showing the permission letter of the project for collecting the samples.

Participants 

There were a total of 96 participants who fulfilled the age criteria of 20 to 40 years and participants who had a unilateral ACL injury and reconstruction with a history of a minimum of five years. After contacting the participants, two groups were formed: Group 1 (42 participants) included individuals doing any kind of physical activity for at least two years postoperatively, and Group 2 (54 participants) included individuals who were living sedentarily after their ACL reconstruction. The sampling technique used was the stratified random sampling method. 

The exercising population was divided into low, moderate, and high-intensity exercises based on the maximum heart rate. We calculated the maximum heart rate by subtracting the age from 220. Moderate-intensity exercises are those exercises that have a target heart rate range between 50 to 70% of the maximum heart rate, whereas high-intensity exercises have a target heart rate range between 70 to 85% of the maximum heart rate. 

Inclusion and exclusion criteria 

Our study included both males and females. Participants were in the age criteria 20-40 years, and they must have a unilateral ACL injury and reconstruction with a history of a minimum of five years. Those participants who had known cases of OA other than ACL injuries were excluded from the study. 

Ethical details 

The research was accepted by the institutional ethical committee of Krishna Vishwa Vidyapeeth, KIMSDU (Protocol No: 155/2023-2024) in Karad, Maharashtra. The Helsinki declaration of 1964 and its subsequent amendments, as well as the ethical requirements of the relevant organizational and national research committees, were followed in all procedures carried out in studies involving human participants.

Assessment tool

Radiographs before surgery or immediate postoperative radiographs versus recent (after five years) X-ray reports were used to assess the arthritic changes in the knee joint and for data collection. A statistical analysis was performed based on the collected data. The radiographs were taken in the standing position. 

**Figure 1:**
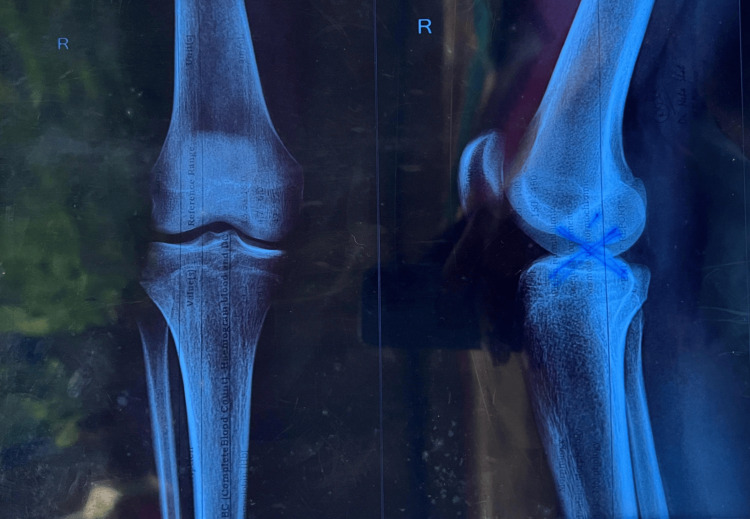
Knee radiograph of a patient taken just before the surgery.

Figure 1 below shows a radiograph of a patient taken just before the surgery. 

**Figure 2:**
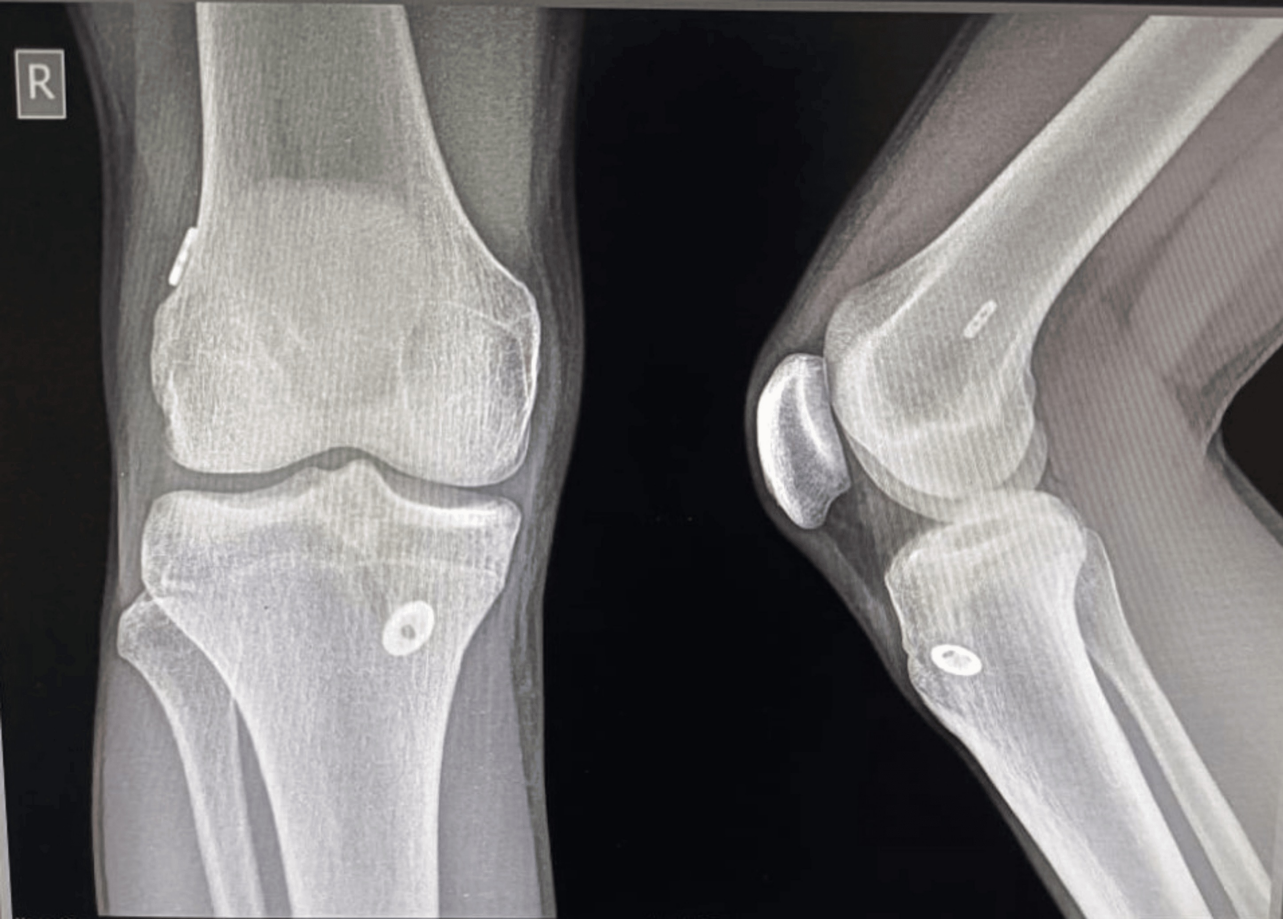
Knee radiograph of the same patient after 5 years

Figure 2 below represents the radiograph of the same patient taken after five years.

Outcome measures

Kellgren and Lawrence classification (KLC) was used to assess the severity of OA in both groups. It was graded from 0 (No presence of OA) to 4 (Severe OA) [14].

Statistical analysis 

Statistical analysis was performed using GraphPad Prism software. Mean and standard deviation (SD) were calculated for each group. An unpaired t-test was used to compare the mean Kellgren-Lawrence grades between the exercising and sedentary groups.

## Results

Table 1 represents demographic data of collected samples who had undergone an ACL reconstruction. Among the exercising participants, 29 (69.04%) were males and 13 (30.95%) were females, whereas among sedentary individuals, 32 (59.25%) were males and 22 (40.74%) were females. Out of the total population, it was found that most of the individuals belonged to age group 21-25, that is, 24 (57.14%) in the exercising and 23 (38.88%) in the non-exercising individuals. The occupation was categorized into different domains like students, athletes, doctors, engineers, farmers, housewives, corporate businessmen, and teachers. Among all of them, students and athletes had greater relevance to an ACL rupture. It was 18 (42.85%) among students and 12 (28.57%) among athletes in the exercising population, whereas it was 20 (37.03%) among students in the sedentary population.

**Table 1:** Demographic data of the population.

Parameters		Frequency	
Age		Exercising	Non-Exercising
	Less than 21	1	1
	21-25	24	21
	26-30	13	16
	31-35	1	9
	36-40	3	7
Gender	Males	29	32
	Females	13	22
Weight	51-60	12	10
	61-70	20	13
	71-80	7	18
	81-90	3	13
Occupation	Corporate	4	11
	Doctor	2	5
	Athlete	12	0
	Housewife	2	7
	Industry worker	2	0
	Student	18	20
	Teacher	0	2
	Engineer	0	5
	Businessmen	2	4

The assessment of post-operative X-rays, as detailed in Table 2, revealed that 39 patients (92.85%) in the exercising population exhibited grade 0 osteoarthritis (OA), while three patients (7.14%) were classified as grade 1, with no patients falling into grades 2 or 3. However, a comparison with recent X-rays demonstrated a significant decrease in the percentage of patients classified as grade 0, which declined to 31 patients (73.80%). In contrast, there was an increase in the number of patients classified in higher grades: grade 1 increased to seven patients (16.66%), grade 2 to 3 patients (7.14%), and grade 3 to 1 patients (2.38%). Statistical analysis showed a significant difference in the mean Kellgren-Lawrence grade between the exercising and sedentary groups (p<0.0001 for both groups). 

**Table 2:** Data regarding number of individuals falling under respective grades using Kellgren and Lawrence classification of knee OA along with statistical analysis.

Grade	Exercising population		Sedentary population	
	Post-op	Recent	Post-op	Recent
0	39	31	41	25
1	3	7	13	19
2	0	3	0	6
3	0	1	0	4
Mean±SD		0.38±0.73		0.79±0.91
p-value		<0.0001		<0.0001
Total	42		54	

The findings regarding the progression of osteoarthritis (OA) in sedentary individuals following anterior cruciate ligament (ACL) surgery reveal significant changes over time. Initially, 41 patients (75.9%) were classified with grade 0 OA, while 13 patients (24.07%) were at grade 1, with no patients in grades 2 or 3 during the initial postoperative X-ray assessment. However, a follow-up comparison with recent X-rays indicated a notable shift: the percentage of patients in grade 0 decreased to 25 (46.29%), while those classified as grade 1 increased to 19 patients (35.18%). Additionally, new classifications were observed, with six patients (11.11%) in grade 2 and four patients (7.40%) in grade 3. This data highlights the progression of arthritic changes in both exercising and sedentary populations following ACL surgery, indicating a significant shift towards higher grades of OA over time, especially in the sedentary population.

**Figure 3:**
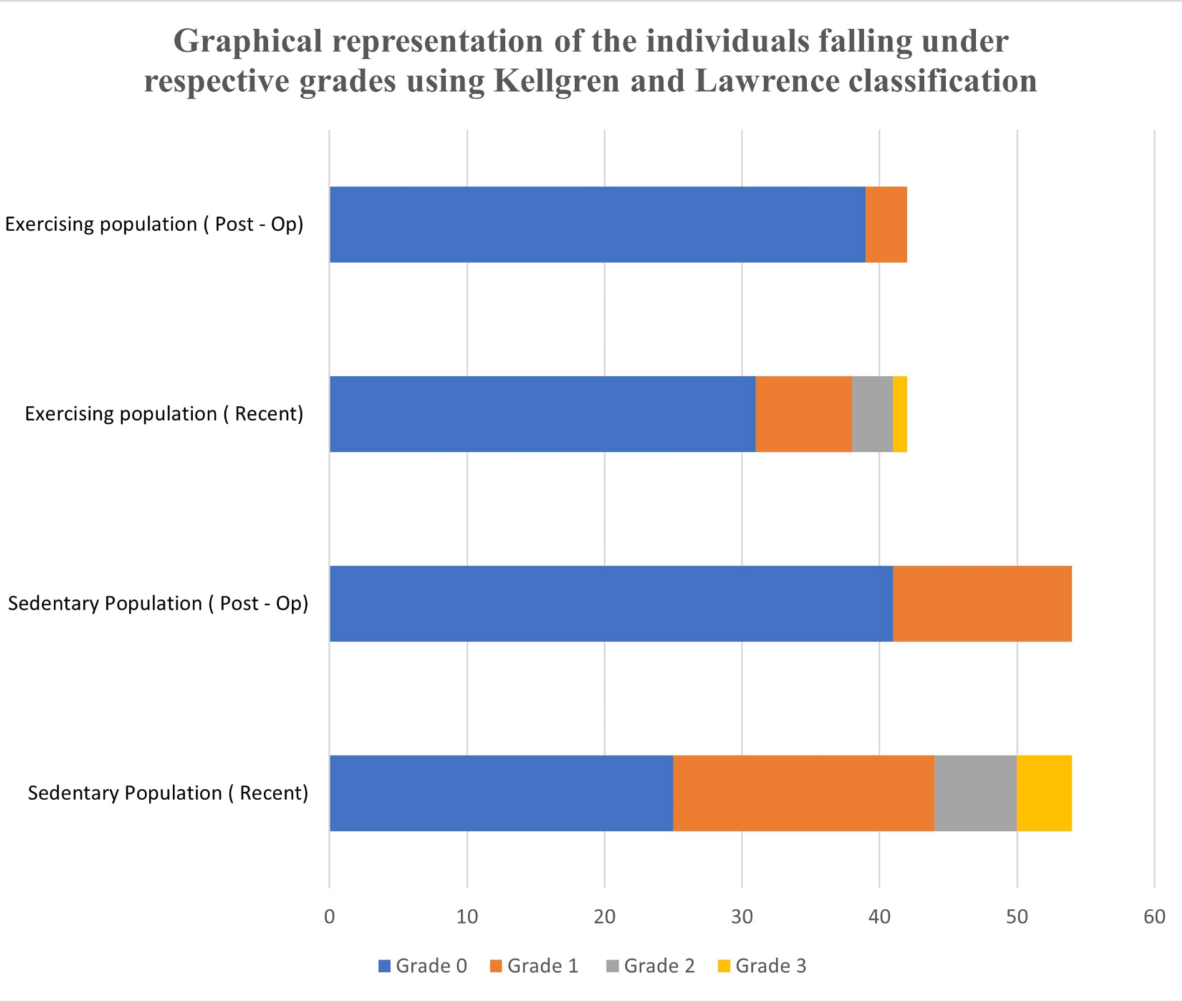
Distribution of Kellgren-Lawrence grades in exercising and sedentary populations (post-op and recent).

Figure 3 below shows a graphical distribution of the population falling under the respective grades using the Kellgren-Lawrence classification. 

Table 3 below indicates that 11 (26.19%), 15 (35.71%), and 16 individuals (38.09%) were involved with low, moderate, and high-intensity exercises, respectively. 

**Table 3:** Type of activities done by the exercising population.

Exercise intensity	Type of activity	Frequency
Low-intensity exercises	Slow walking	7
	Yoga and pilates	4
Moderate-intensity exercises	Brisk walking/jogging	6
	Cycling	5
	Resistance training	4
High-intensity exercises	Football	10
	Basketball	4
	Running	6

**Figure 4:**
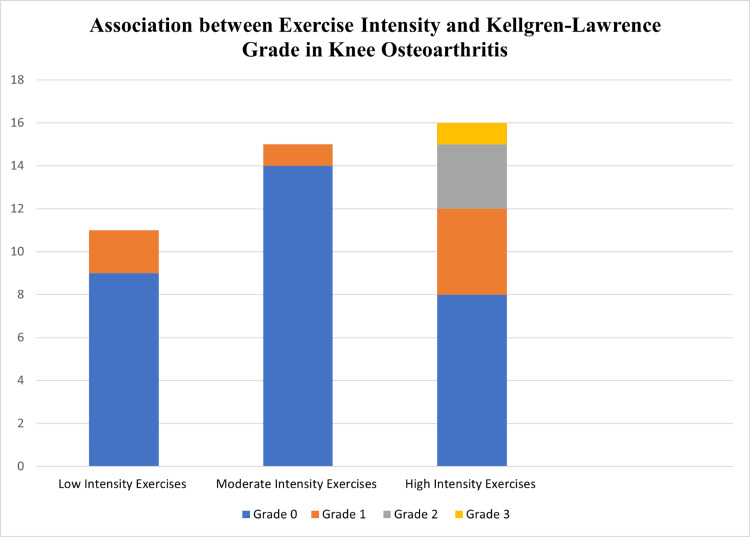
Association between exercise intensity and Kellgren-Lawrence grade in knee osteoarthritis.

The exercising population was divided into individuals engaged with low, moderate and high-intensity exercises after their ACL reconstruction. Figure 4 below represents the association of the exercise population with Kellgren and Lawrence classification. Participants engaged in low or moderate-intensity exercises demonstrated no signs of radiographic osteoarthritis. Conversely, the high-intensity exercise group displayed evidence of osteoarthritis in three individuals (grade 2) and one individual (grade 3). 

As presented in Table 4, the prevalence of arthritic changes was observed in four patients (9.52%) from the exercising population and 10 patients (18.51%) from the sedentary population. 

**Table 4:** Number of patients who had undergone arthritic changes in exercising and sedentary population.

Population type	OA present	OA absent
Exercising	4	38
Non-exercising	10	44

## Discussion

This was a prevalence-based study aimed to find prevalence of arthritic changes in the exercising and non-exercising post operative ACL injury patients. In this study, we aimed to find the occurrence of arthritic changes in the knee in exercising and sedentary individuals after ACL reconstruction.

In this study, the age group 21-30 was common because most of the individuals who underwent an ACL reconstruction were students and athletes. The reason behind this might be due to the high level of participants being involved in physical activity and sports, especially in this age group. In a study by Joshi A et al., most injuries occurred in the 15-30 years age group as sports were the most common cause of ACL injury in patients below 30 years of age, whereas road traffic accidents (RTAs) were the most common cause for ACL injuries above 30 years of age [15].

Although men participated in leisure sports at a higher rate than women did in our study, men experienced more ACL injuries. However, a study by F. Mancino et al. disputes our assertion and claims that women are more likely than men to sustain ACL damage. The laxity of the ligament and the general alignment of the limbs are directly affected by frequent shifts or fluctuations in the level of estrogen [16]. Also, another study by Griffin et al. concluded that females are at an increased risk of ACL injuries than males due to proposed anatomical risk factors such as increased Q-angle, narrower femoral notch, and increased hypermobility or laxity in female athletes [17].

In the exercising population, lower rates of arthritic changes were found than in the sedentary population. While individuals have transitioned from grade 0 to grade 1 in the exercising population after assessing their recent X-ray, the rate of progress was slower in comparison to those who are sedentary, as seen from Table 2 in the results. Moderate-intensity exercise has been shown to decrease exacerbating symptoms of OA, as concluded in a study by Xiaofeng Deng et al., which says that moderate exercise helps prevent OA pathogenesis by various mechanisms such as cell death, cell programming, and autophagy. This shows that after ACL surgery, regular exercise seems to lower the risk of arthritis developing in the injured knee [18]. Moreover, individuals performing high-intensity exercises in our study were prone to arthritic changes in their knees, but the magnitude was lower when compared with the sedentary population. High-intensity exercises are known to increase mechanical loading on the knee joint, causing degradation of the articular cartilage. A study by Yahong Wu et al. coincides with our findings and indicates that participants doing higher mechanical strain activity were at an increased risk of knee osteoarthritis [19]. 

A slightly higher rate of arthritic changes was seen in the sedentary group due to a complete lack of exercise or physical activity. Higher progression rates were observed from grade 0 to grade 1 in the sedentary population and the subsequent grades, highlighting the importance of physical activity. Most of the sedentary population was involved with increased sitting and standing hours, increasing stress on the knee joint. A study by Huang et al. confirmed that prolonged sitting can cause decreased joint stability due to a reduction in muscle strength, further accelerating cartilage degeneration [20]. One more study by Abdulrahman A. Aldosari et al. showed that very to extremely severe knee OA was significantly associated with a low level of physical activity among male and female patients. It concluded that most of the study patients had severe to extremely severe knee OA with low physical activity levels [21]. 

Limitations

The limitation of this study is the reliance solely on radiographic assessment for the evaluation of knee osteoarthritis (OA). Clinical assessments, such as the Western Ontario and McMaster Universities osteoarthritis index (WOMAC) or the knee injury and osteoarthritis outcome score 1 (KOOS), could have provided additional information about the patient's condition. Also, our sample size was low and had a limited geographic scope, which could affect the generalized results. 

## Conclusions

The study has found a low level of prevalence of arthritic changes in exercising post-operative ACL injury individuals when compared to sedentary post-operative ACL injury individuals. Moderate-intensity exercises have been shown to decrease the occurrence of arthritic changes in the exercising population, whereas the sedentary population has a slightly higher rate of those changes. These findings can profoundly impact the population on the importance of exercise in preventing knee OA, particularly after an ACL reconstruction. Future studies can focus on providing more information specifically on the type of exercise programs (resistance or endurance-based) responsible for preventing knee osteoarthritis in the long term, which can be of great significance, given the increasing rate of ACL injuries in the younger population. 
